# Prescription opioids, alcohol and fatal motor vehicle crashes: a population-based case-control study

**DOI:** 10.1186/s40621-019-0187-x

**Published:** 2019-03-25

**Authors:** Guohua Li, Stanford Chihuri

**Affiliations:** 1 Center for Injury Epidemiology and Prevention, Columbia University Irving Medical Center, 622 West 168th St, PH5-505, New York, NY 10032 USA; 2 Department of Anesthesiology, Columbia University College of Physicians and Surgeons, 622 West 168th St, PH5-505, New York, NY 10032 USA; 3 Department of Epidemiology, Columbia University Mailman School of Public Health, 622 West 168th St, PH5-505, New York, NY 10032 USA

**Keywords:** Alcohol, Driving safety, Interaction, Motor vehicle crashes, Opioid epidemic, Prescription opioids, Polydrug

## Abstract

**Background:**

The prevalence of prescription opioid use among drivers has increased markedly in the past two decades. The purpose of this study is to assess the associations of prescription opioid use and alcohol use with the risk of fatal crash involvement in US drivers.

**Methods:**

We performed a population-based case-control study using toxicological testing data from two national data systems. Cases (*n* = 3606) were drivers involved in fatal motor vehicle crashes selected from the Fatality Analysis Reporting System and controls (*n* = 15,600) were drivers participating in the 2007 and 2013 National Roadside Surveys of Alcohol and Drug Use by Drivers. Multivariable logistic regression was used to estimate odds ratios (ORs) and 95% confidence intervals (95% CIs) of fatal crash involvement associated with prescription opioid use with and without the presence of alcohol.

**Results:**

Overall, cases were significantly more likely than controls to test positive for prescription opioids (5.0% vs. 3.7%, *p* < 0.001), alcohol (56.2% vs. 7.1%, *p* < 0.0001), and both substances (2.2% vs. 0.2%, *p* < 0.001). Relative to drivers testing negative for prescription opioids and alcohol, the adjusted ORs of fatal crash involvement were 1.72 (95% CI: 1.37, 2.17) for those testing positive for prescription opioids and negative for alcohol, 17.92 (95% CI: 16.19, 19.84) for those testing positive for alcohol and negative for prescription opioids, and 21.89 (95% CI: 14.38, 33.32) for those testing positive for both substances. The interaction effect on fatal crash risk of prescription opioid use and alcohol use was not statistically significant on either additive or multiplicative scale.

**Conclusions:**

Prescription opioid use is associated with a significantly increased risk of fatal crash involvement independently of alcohol use. Concurrent use of prescription opioids and alcohol is associated with a 21-fold increased risk of fatal crash involvement.

## Background

Prescription opioids, such as oxycodone and hydrocodone, are widely used in pain management and as anesthetics in surgery (Manchikanti and Singh 2008; Han et al. 2017; Governor’s Highway Safety Association (GHSA), 2018). In 2015, nearly 92 million US adults (about 38% of the total adult population) reported taking a legitimately prescribed opioid (Han et al. 2017). In light of unabated increases in drug overdose mortality, the US federal government declared the opioid epidemic a national public health emergency in October 2017. Although the opioid epidemic is viewed primarily through counts of overdose fatalities, its impact on driving safety has become a cause of concern. Motor vehicle crashes are the second leading cause of unintentional injury mortality, and death rates per 100 million vehicle miles traveled have increased 2.6% from 2015 to 2016 following a long downward trend (National Center for Injury Prevention and Control 2016; National Highway Traffic Safety Administration 2017). While annual prescriptions of opioids have decreased from 277 million in 2012 to 239 million in 2016, the prevalence of prescription opioids detected among fatally injured drivers continues to rise (Brady and Li 2012; Pezalla et al. 2017; Chihuri and Li 2017a; Faryar et al. 2018). In 2016, 10.7% of all fatally injured drivers tested positive for prescription opioids, representing a 10-fold increase since 1995 (Chihuri and Li 2017a; Governor’s Highway Safety Association (GHSA), 2018).

Use of prescription opioids may impair psychomotor and cognitive skills necessary for safe operation of a motor vehicle, such as manual dexterity, hand-eye coordination, mental alertness, and visual information processing (Office of National Drug Control Policy 2011; Dowell et al. 2016; Ferreira et al. 2018). While results of experimental studies examining the effects of prescription opioid use on driving performance are inconsistent (Lenné et al., 2000; Fishbain et al. 2003; Kress and Kraft 2005; Borgeat 2010; Leung 2011; Strand et al. 2016; Strand et al. 2017), it is evident that drivers on prescription opioids tend to show decreased visual information processing speed and accuracy (Gaertner et al. 2006; Byas-Smith et al. 2005; Mailis-Gagnon et al. 2012). Similarly, findings from epidemiological studies are conflicting, with most studies reporting significantly increased risks of crash involvement and crash culpability associated with prescription opioid use (Bruera et al. 1989; Monárrez-Espino et al., 2013; Gjerde et al. 2015; Rudisill et al. 2016; Chihuri and Li 2017b, 2019) and others reporting no evidence of increased risk (Bachs et al. 2009; Ray et al., 1992; Sims et al., 1998; Dussault et al., 2002). These inconsistences are likely caused in part by the increasing prevalence of prescription opioid use in drivers and differences in research methods and study populations. For example, some studies included only younger drivers (Orriols et al. 2009), or only older drivers (Marquet et al. 1998), while other studies were restricted largely to male drivers (Monárrez-Espino et al., 2016) or apparently underpowered (Christensen et al. 1990). Polydrug use involving prescription opioids and the interaction of alcohol and prescription opioids are of increasing concern given that over 20% of fatally injured drivers test positive for two or more drugs (Movig et al. 2004; Drummer et al. 2004). The purpose of this study is to assess the associations of prescription opioid use and alcohol use with the risk of fatal motor vehicle crash involvement.

## Methods

### Data sources

We used data from two national surveillance systems maintained by the National Highway Traffic Safety Administration (NHTSA, Washington, DC): 1) the Fatality Analysis Reporting System (FARS) and 2) the 2007 and 2013–14 National Roadside Survey of Alcohol and Drug Use by Drivers (NRS). FARS is an annual census of motor vehicle crashes occurring on US public roadways that result in at least one fatality within 30 days in all 50 states, the District of Columbia and Puerto Rico (Li et al. 2013; National Highway Traffic Safety Administration 2016). For each crash, the FARS records detailed data on crash circumstances (e.g., date, time, road and weather conditions), individuals involved (e.g., driver, passenger, pedestrian, and cyclist), driver characteristics (e.g., age, sex, race, drug testing results, driving history within the previous 3 years, survival status) and vehicle characteristics (e.g., vehicle type, make, model and year). Data in the FARS come from multiple sources, including the coroner or medical examiners’ reports, state highway patrol records, police accident reports, death certificates, medical records and vehicle registration files. Analysts improve the accuracy and completeness of data through automatic checks of each entry and other rigorous quality control programs. Although FARS was incepted in 1975, collection of toxicological testing data for nonalcohol drugs started in 1991 (National Highway Traffic Safety Administration 2016).

The 2007 and 2013–14 NRSs were national field surveys to estimate the prevalence of alcohol and drug use in the driver population. The surveys involved randomly stopped and verbally consented non-commercial drivers at 300 locations across the continental United States (Lacey et al. 2009; National Highway Traffic Safety Administration 2010a; Kelley-Baker et al. 2016). Drivers were selected through a four-stage stratified random sampling scheme based on primary sampling units, police jurisdictions, survey locations and passing-by drivers. Drivers who passed through designated locations were randomly stopped and invited to participate in the surveys. Commercial vehicle drivers, drivers under the age of 16, and drivers who could not communicate in English or Spanish were excluded from the surveys. Eligible drivers who voluntarily participated in the surveys were asked to provide an oral fluid sample for drug testing and a breath sample for alcohol testing. Participating drivers also provided additional information on demographic characteristics, self-reported drinking and drug use behavior, mileage and trip origin and destination. Both the 2007 and 2013–14 NRS surveys were conducted from 9:30 am to 11:30 am and 1:30 pm to 3:30 pm on Fridays and from 10 pm to midnight and 1 am to 3 am on both Friday and Saturday nights. The 2007 survey was conducted from July 20 through December 12, 2007 and the 2013–14 survey from June 7, 2013 through March 30, 2014. Of the 22,009 eligible drivers (10,909 for the 2007 NRS and 11,100 for 20013–14 NRS), 15,600 (70.9%; 70.8% for the 2007 NRS and 71.0% for the 2013–14 NRS) completed the interview and provided an oral fluid sample for drug testing (including 7905 drivers who also provided a blood sample). The sampling methods and study protocols for the 2007 and 2013–14 NRSs were described in detail elsewhere (Lacey et al. 2009; Kelley-Baker et al. 2016).

### Study design

We used a population-based case-control study design to assess the individual and joint effects of prescription opioid use and alcohol use on the risk of motor vehicle crash involvement. Of the 6783 eligible cases, 3177 (46.8%) were excluded from the analysis because of missing toxicological testing data [including 262 drivers involved in fatal crashes in Maryland, New Mexico, and North Carolina due to unreliable drug testing data recorded in the FARS from these states (Drummer et al. 2004; Brady and Li 2012; Chen et al. 2018)]. Consequently, cases included in the study were 3606 drivers who were involved in fatal motor vehicle crashes at specific times from 2006 to 2008 and from 2012 to 2014 and for whom toxicological testing data were available. Controls included in the study were 15,600 drivers who participated in the 2007 NRS or the 2013–14 NRS. Cases and controls were matched on time of day, day of week, and month of year (i.e., from 9:30 am to 11:30 am and 1:30 pm to 3:30 pm on Fridays and from 10 pm to midnight and 1 am to 3 am on both Friday and Saturday nights). The Columbia University Irving Medical Center Institutional Review Board (New York, NY) deemed this study exempt from review under 45 CFR 46.

### Drug testing assessments

Toxicological tests for cases were based on blood and/or urine specimens through radioimmunoassay techniques and liquid/gas chromatography combined with mass spectrometry (Li et al. 2013; Kelley-Baker et al. 2016). Of all cases, 3247 (90.0%) had at least one drug test based on blood specimens. For each case, up to 3 nonalcohol drugs were recorded in the FARS. When multiple nonalcohol drugs were detected, drugs were recorded in the following order: narcotics, depressants, stimulants, marijuana, and other drugs (National Highway Traffic Safety Administration 2010b). Prescription opioids fall under narcotics and are therefore recorded first when there are multiple drugs. If a drug and its metabolite were detected, only the parent drug was recorded. Prescription drugs were coded according to the FARS coding manual and included injectable or oral formulations of hydrocodone, meperidine, oxycodone, morphine, codeine, fentanyl, oxymorphone, propoxyphene, hydromorphone, diphenoxylate, methadone, and oxymorphone. Illegal opioids such as heroin were excluded from the analysis. Blood alcohol concentrations (BACs) were measured in grams per deciliter where a BAC of 0.01 g/dl or greater was considered alcohol-positive. Toxicological tests for controls were based on oral fluid samples, first through the enzyme-linked immunosorbent assay technique to screen for the presence of drugs and if positive, then through liquid/gas chromatography combined with mass spectrometry for confirmation (Lacey et al. 2009; National Highway Traffic Safety Administration 2010b; Kelley-Baker et al. 2016). Of the 15,600 drivers in the control group, 7905 (50.7%) also provided blood samples for drug testing. For most prescription opioids, the minimum detection concentration was 20 ng/ml in oral fluid screening, 25 ng/ml in blood screening and 10 ng/ml for both oral and blood confirmation (Lacey et al. 2009; Kelley-Baker et al. 2016). A sample was regarded as positive if the minimum threshold was attained during the confirmation test; otherwise it was regarded as negative. Common prescription opioids detected in controls included codeine, morphine, hydrocodone, hydromorphone, methadone and meperidine. BACs for controls were determined from samples measured using the evidential breath test device.

### Statistical analyses

We estimated odds ratios (ORs) and 95% confidence intervals (95% CIs) through logistic regression modeling. The potential interaction effect of prescription opioid use and alcohol use on the risk of fatal crash involvement was assessed using drivers who tested negative for both prescription opioids and alcohol as the reference group. Crude ORs and 95% CIs were computed according to driver age, sex, geographic region, prescription opioid testing results and BACs. BAC data were analyzed as a binary variable (positive if BAC ≥ 0.01 g/dl and negative if BAC < 0.01 g/dl) as well as a 3-level category variable (BAC < 0.01, 0.01–0.07, and ≥ 0.08 g/dl). Additive interaction was assessed based on three measures; the relative excessive risk due to interaction (RERI), the attributable proportion due to interaction (AP) and the synergy index (S) as follows:

$$ RERI={OR}_{opioids+ alcohol}-{OR}_{opioids}-{OR}_{alcohol}+1 $$

$$ AP= RERI/\left({OR}_{opioids+ alcohol}\right) $$

$$ S=\left[{OR}_{opioids+ alcohol}-1\right]/\left[\left({OR}_{opioids}-1\right)+\left({OR}_{alcohol}-1\right)\right] $$

where RERI = 0, AP =0, and S = 1 denote absence of interaction on the additive scale. Estimates for RERI, AP, S and corresponding 95% confidence intervals were computed using the method of variance estimates recovery (Zou 2008), and the asymptotic approach (Hosmer and Lemeshow 1992). Estimates for RERI were also verified using an approach proposed by Knol and VanderWeele (2012). In addition, interaction on the multiplicative scale was assessed through the prescription opioids-alcohol interaction term in the multivariable logistic regression model.

To assess robustness of our findings, we performed five sets of sensitivity analyses. First, we included cases and controls with toxicological testing results from blood samples only. Second, we restricted the analysis to cases from 12 states (CA, CO, CT, MA, NH, NY, OH, PA, RI, VT, WA, WV) that tested more than 80% of fatally injured drivers during the study period (Brady and Li 2012). Third, we performed an analysis based on weighted data for the controls to account for the complex survey design and nonparticipating drivers. Fourth, we performed a stratification analysis by dividing the data into two time periods according to the 2007 and 2013–14 NRSs. Finally, we restricted the analysis to cases that died at the crash scene to minimize the possible bias introduced by opioid analgesics used after crashes for trauma care and management. To assess the extent to which unmeasured confounding might bias the results, we computed the E-value statistic (VanderWeele and Ding, 2017). All analyses were performed using SAS 9.4 (SAS Institute Inc., Cary, NC).

## Results

Cases included in the study were similar to those eligible drivers excluded from the analysis due to missing toxicological testing data in crash circumstances, but were younger (mean age = 38.6 ± 28.4 years vs. 41.9 ± 30.9 years, *p* < 0.0001), more likely to be male (80.8% vs. 77.9%, *p* = 0.0034), more likely to have had a license suspension within the previous 3 years (59.1% vs. 51.9%, *p* < 0.0001), and more likely to be involved in nighttime crashes (68.4% vs. 62.3%, *p* < 0.0001).

Compared to controls, cases were slightly older, more likely to be male and were more likely to be from the Southern or Northeast regions (Table 1). Of the cases, 56.2% tested positive for alcohol and 5.0% positive for prescription opioids, compared with 7.1 and 3.7% of controls, respectively (Table 1). Among the cases testing positive for alcohol, 87.5% had BACs ≥0.08 g/dl, compared with 19.7% of the alcohol-positive controls. On the bivariable level, significantly increased odds ratios of fatal crash involvement were associated with older age (≥ 65 years), male sex, the Southern region, elevated BACs, and use of prescription opioids (Table 1).

**Table 1:** Crude Odds Ratios (ORs) and 95% Confidence Intervals (CIs) of Fatal Crash Involvement according to Driver Characteristics in the Continental United States, Selected Time Periods on Fridays and Saturdays, July 20 through December 1, 2006, 2007, 2008, and June 7, 2012 through March 30, 2013 and June 7, 2013 through March 30, 2014

Driver Characteristics^a^	Cases, %	Controls, %	Crude OR	95% CI
	(*n* = 3606)	(*n* = 15,600)		
Age, years				
16–24	27.7	28.6	1.04	0.95, 1.14
25–39	32.0	32.6	1.05	1.00, 1.15
40–64	31.3	33.4	1.00	reference
≥ 65	9.0	5.4	1.80	1.56, 2.07
Sex				
Female	19.2	40.8	1.00	reference
Male	80.8	59.2	2.90	2.65, 3.17
Region				
West	22.2	22.5	1.00	reference
Midwest	22.6	27.9	0.82	0.73, 0.91
Southern	38.4	33.9	1.14	1.04, 1.26
Northeast	16.8	15.7	1.08	0.99, 1.21
Blood alcohol concentration (BAC, g/dl)				
<0.01	43.8	92.9	1.00	reference
0.01–0.07	7.0	5.7	2.63	2.27, 3.06
≥ 0.08	49.2	1.4	74.72	64.31, 86.81
Prescription opioids				
Negative	95.0	96.3	1.00	reference
Positive	5.0	3.7	1.36	1.14, 1.61

^a^There were 52 cases with missing data on blood alcohol concentrations

There were 126 controls with missing data on sex, 297 on age, 7 on blood alcohol concentrations

After adjusting for age, sex, region, and BAC level, use of prescription opioids was associated with a 71% increased risk of fatal crash involvement (adjusted OR = 1.71, 95% CI: 1.37, 2.14) (Table 2). As expected, substantially increased ORs were associated with elevated BACs, particularly for drivers with BACs ≥0.08 g/dl) (Table 2).

**Table 2:** Adjusted Odds Ratios (ORs) and 95% Confidence Intervals (CIs) of Fatal Crash Involvement according to Driver Characteristics in the Continental United States, Selected Time Periods on Fridays and Saturdays, July 20 through December 1, 2006, 2007, 2008, and June 7, 2012 through March 30, 2013 and June 7, 2013 through March 30, 2014

Driver characteristic	Adjusted OR	95% CI
Age, years		
16–24	0.85	0.74, 0.96
25–39	0.68	0.60, 0.78
40–64	1.00	reference
≥ 65	2.81	2.37, 3.33
Sex		
Female	1.00	reference
Male	2.06	1.84, 2.31
Region		
West	1.00	reference
Midwest	0.81	0.70, 0.94
Southern	1.06	0.93, 1.21
Northeast	0.70	0.59, 0.83
Blood alcohol concentration (BAC, g/dl)		
<0.01	1.00	reference
0.01–0.07	2.59	2.20, 3.06
≥ 0.08	80.09	68.21, 94.04
Prescription opioids		
Negative	1.00	reference
Positive	1.71	1.37, 2.14

**Fig. 1:**
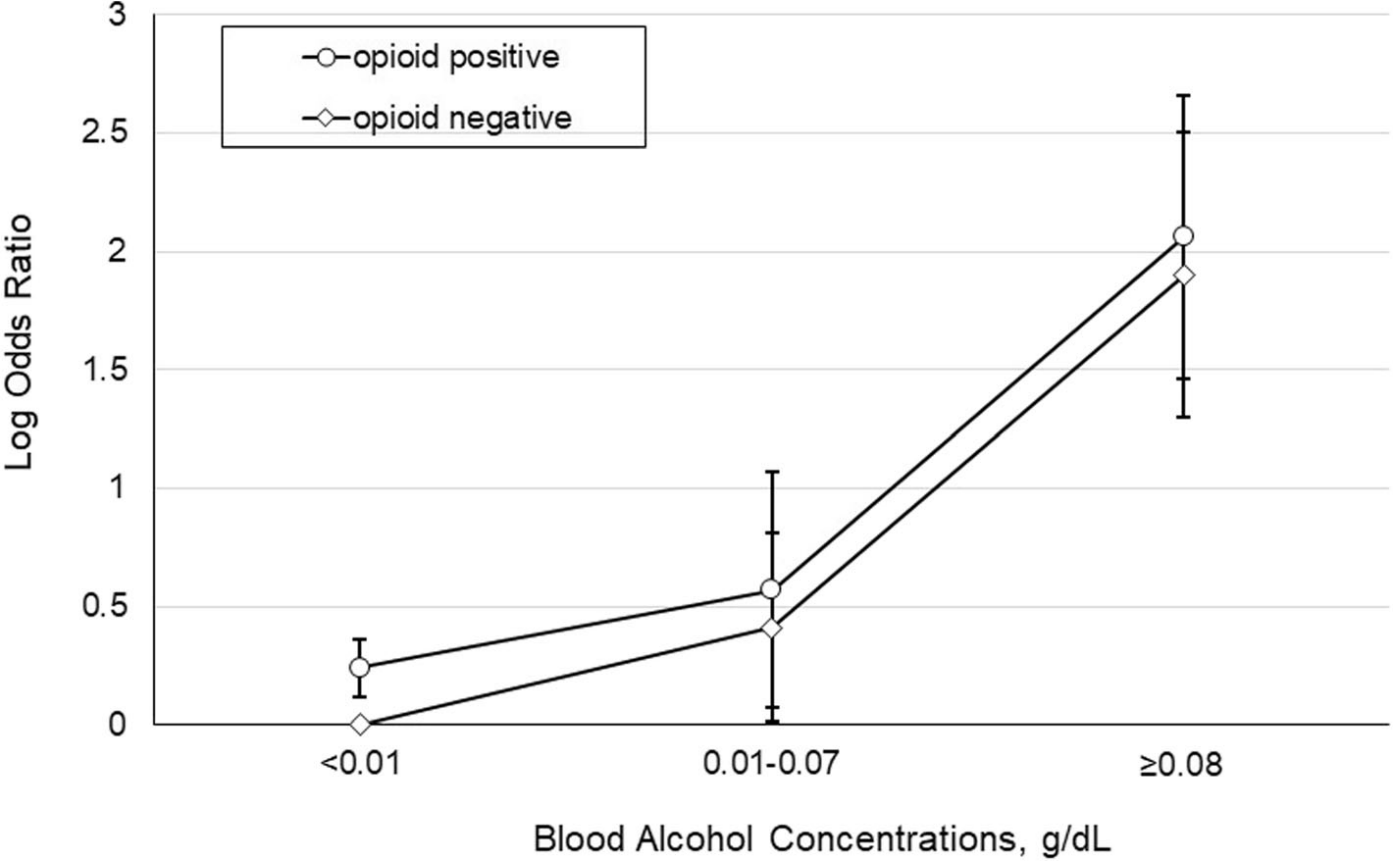
Estimated Adjusted Odds Ratios of Fatal Crash Involvement According to Blood Alcohol Concentrations and Prescription Opioid Testing Result

A total of 79 (2.2%) cases and 31 (0.2%) controls tested positive for both prescription opioids and alcohol. Relative to drivers who tested negative for both prescription opioids and alcohol, the estimated odds of fatal crash involvement increased 72% for those testing positive for prescription opioids and negative for alcohol, nearly 17-fold for those testing negative for prescription opioids and positive for alcohol, and about 21-fold for that testing positive for both prescription opioids and alcohol (Table 3). The interaction term indicating concurrent use of prescription opioids and alcohol was not statistically significant (χ^2^_(df = 1)_ = 1.8337, *p* = 0.1757). None of the three statistics for measuring interaction on the additive scale were significant in the unadjusted model (RERI = 3.63, 95% CI: -5.4, 17.42; AP = 0.14: 95% CI: -0.34, 0.44; S = 1.18, 95% CI 0.74, 1.86) and in the adjusted model (RERI = 3.47, 95% CI: -3.96, 14.50; AP = 0.16: 95% CI: -0.28, 0.43; S = 1.20, 95% CI 0.78, 1.84). The risk of fatal crash involvement increased with BACs in parallel between drivers testing negative for prescription opioids and those testing positive (Fig. 1).

**Table 3:** Estimated Odds Ratios (ORs) and 95% Confidence Intervals (CIs) of Fatal Crash Involvement According to Driver Prescription Opioid and Alcohol Testing Results in the Continental United States, Selected Time Periods on Fridays and Saturdays, July 20 through December 1, 2006, 2007, 2008, June 7, 2012 through March 30, 2013 and June 7, 2013 through March 30, 2014

Testing Result		Crude OR	95% CI	Adjusted OR^a^	95% CI
Opioids	Alcohol				
Negative	Negative	1.00	Reference	1.00	Reference
Positive	Negative	1.66	1.33, 2.08	1.72	1.37, 2.17
Negative	Positive	17.12	15.62, 18.77	17.92	16.19, 19.84
Positive	Positive	22.31	1.99, 7.68	21.89	14.38, 33.32

^a^Adjusted for age, sex and region

Results from sensitivity analyses showed significantly elevated ORs of fatal crash involvement associated with prescription opioid use when adjusting for alcohol, age, sex, and region. Specifically, the estimated adjusted ORs were 1.45 (95% CI: 1.12, 1.87) when restricted to blood-based samples only, 1.68 (95% CI: 1.15, 2.45) when restricted to states that tested 80% or more of fatally injured drivers, 1.78 (95% CI: 1.37, 1.2.31) when weighted data for controls were used, 1.61 (95% CI: 1.13, 2.31) when study period was restricted to 2006–2008,1.79 (95% CI: 1.33, 2.47) when study period was restricted to 2012–2014, and 1.89 (95% CI: 1.44, 2.49) when restricted to cases that died at the crash scene. Results from all sets of sensitivity analyses indicate the absence of significant interaction effects between prescription opioid use and alcohol on the risk of fatal crash involvement on either multiplicative or additive scale. The estimated E-value was 2.83 (95%CI: 2.08,3.58), which would be the minimum OR of fatal crash involvement associated with an unmeasured confounding necessary to completely explain away the association between prescription opioid use and the risk of fatal crash involvement observed in this study.

## Discussion

Results of this study indicate that prescription opioid use by drivers is associated with a significantly elevated risk of fatal crash involvement, independently of alcohol use. Our results add more evidence to the existent literature about the effects of prescription opioids on driving safety (Chihuri and Li 2017b). In light of the increasing prevalence of prescription opioid use among drivers, our finding suggests that it is urgent to better understand and control the impact of the ongoing opioid epidemic on drugged driving and related injury morbidity and mortality from motor vehicle crashes.

This study also reaffirms the important role of alcohol-impaired driving in fatal motor vehicle crashes. Although we did not find a significant synergistic effect between prescription opioids and alcohol on fatal crash risk, concurrent use of these two substances remains cause of concern as 46.4% of the drivers involved in fatal motor vehicle crashes who tested positive for prescription opioids had elevated BACs. Our results reveal that the estimated odds of fatal crash involvement for drivers using both prescription opioids and alcohol are nearly 22 times that for those using neither of the substances.

Driving under the influence of drugs (DUID), such as marijuana and opiates, is prohibited in all US states and the District of Columbia. Enforcement of DUID laws, however, has been hindered by the lack of portable, affordable, and noninvasive technologies for rapid and accurate drug testing and inadequate research evidence (Government Accountability Office 2015). To reduce the adverse impact of the opioid epidemic on traffic injuries and fatalities, it is necessary to increase public awareness about the hazards of prescription opioids posed to driving safety through enhanced education programs, particularly for prescribing clinicians and patients on pain medications. Current practice guidelines state that prescribing clinicians should start at the lowest possible effective dosage and use additional caution when initiating opioids for patients 65 years or older or when increasing dosages (Centers for Disease Control and Prevention 2016). Before initiation of prescription opioids, clinicians should discuss all known benefits and risks including potential risk of motor vehicle crashes with their patients. This is important because approximately 1 in 6 patients on prescription opioids becomes opioid-dependent (Alghnam and Castillo 2017) and because driving while on prescription opioids is a significant risk factor for fatal motor vehicle crashes.

This study has several limitations. First, data on the concentration of prescription opioids detected is unavailable in the FARS data and therefore we are unable to assess the dose-response effect and to establish a possible threshold in morphine milligram equivalents above which crash risk may increase significantly. Second, testing positive for prescription opioids indicates prescription opioid use but not necessarily driving impairment or crash causation induced by prescription opioids. The detection window for prescription opioids in blood is less than 24 h and 1 to 4 days in urine, and the half-life of opioids is relatively short (less than 4 h) (Smith 2009; American Institute of Toxicology Laboratories 2011). Hence, a positive test can be used as an indicator of recent use although we are unable to distinguish whether the use was legal or not. Third, drug testing results were unavailable for 46.9% of the eligible cases, which may introduce selection bias to our results. However, a sensitivity analysis based on cases from 12 states (CA, CO, CT, MA, NH, NY, OH, PA, RI, VT, WA, WV) that tested at least 80% of all fatally injured drivers produced results comparable to our main findings. Fourth, cases included in the study were more likely than those excluded to have a license suspension and to be involved in nighttime crashes, which may lead to overestimation of the odds ratios. However, the observed association between prescription opioid use and the risk of fatal crash involvement is unlikely to be explained by any unmeasured confounding factor given the large E-value (2.83). Finally, the absence of a significant interaction effect between prescription opioid use and alcohol use on the risk of fatal crash involvement should be viewed as a preliminary finding because our study is likely underpowered for assessing a modest or small size of the interaction effect between prescription opioids and alcohol on fatal crash risk. Experimental studies have elicited multiple pathways through which opioids and alcohol may interact biologically, such as by inhibiting or potentiating common enzyme systems (Bodd et al. 1986; Meskar et al. 2001; Callahan et al. 2004). Future research with more refined exposure measurement and larger sample sizes may help advance our understanding of opioid-alcohol interaction on driving safety.

## Conclusion

Driver use of prescription opioids is associated with a 72% increase in the risk of fatal crash involvement. Although prescription opioids and alcohol do not appear to confer a significant synergistic effect, concurrent use of the two substances could increase the risk of fatal crash involvement by nearly 21-fold. Government responses to the opioid crisis should include efforts to understand and reduce drugged driving and related injury morbidity and mortality.

## Abbreviations

BAC: Blood Alcohol Concentration
CI: Confidence Interval
DUID: Driving Under the Influence of Drugs
FARS: Fatality Analysis Reporting System
NHTSA: National Highway Traffic Safety Administration
NRS: National Roadside Survey of Alcohol and Drug Use by Drivers
OR: Odds Ratio
